# A New Integrated Approach to Taxonomy: The Fusion of Molecular and Morphological Systematics with Type Material in Benthic Foraminifera

**DOI:** 10.1371/journal.pone.0158754

**Published:** 2016-07-07

**Authors:** Angela Roberts, William Austin, Katharine Evans, Clare Bird, Magali Schweizer, Kate Darling

**Affiliations:** 1 School of Geography and Geosciences, University of St Andrews, St Andrews, Fife, Scotland, KY16 9AL, United Kingdom; 2 Scottish Association for Marine Science, Scottish Marine Institute, Oban, Argyll, Scotland, PA37 1QA, United Kingdom; 3 School of Geosciences, University of Edinburgh, West Mains Road, Edinburgh, EH9 3JW, United Kingdom; 4 UMR CNRS 6112, LPG-BIAF Recent and Fossil Bio-Indicators, University of Angers, 2 bd Lavoisier, 49045, Angers Cedex, France; University of Innsbruck, AUSTRIA

## Abstract

A robust and consistent taxonomy underpins the use of fossil material in palaeoenvironmental research and long-term assessment of biodiversity. This study presents a new integrated taxonomic protocol for benthic foraminifera by unequivocally reconciling the traditional taxonomic name to a specific genetic type. To implement this protocol, a fragment of the small subunit ribosomal RNA (SSU rRNA) gene is used in combination with 16 quantitative morphometric variables to fully characterise the benthic foraminiferal species concept of *Elphidium williamsoni* Haynes, 1973. A combination of live contemporary topotypic specimens, original type specimens and specimens of genetic outliers were utilised in this study. Through a series of multivariate statistical tests we illustrate that genetically characterised topotype specimens are morphologically congruent with both the holotype and paratype specimens of *E*. *williamsoni* Haynes, 1973. We present the first clear link between morphologically characterised type material and the unique SSU rRNA genetic type of *E*. *williamsoni*. This example provides a standard framework for the benthic foraminifera which bridges the current discontinuity between molecular and morphological lines of evidence, allowing integration with the traditional Linnaean roots of nomenclature to offer a new prospect for taxonomic stability.

## Introduction

The first formal classification system of the foraminifera was proposed in 1826 by d’Orbigny, and since then their identification and delineation as distinct species has been the subject of continued and active enquiry. Despite, or perhaps because of, numerous taxonomic studies spanning nearly 200 years, the current status of benthic foraminiferal taxonomy might be perceived as one of extreme confusion. For example, an estimated 10–25% of modern benthic foraminiferal names have been suggested to be synonyms [1]. Traditionally, specimens of benthic foraminifera have been classified based on a comparative assessment of differences in morphological characteristics. These morphological species concepts are constructed around name-bearing type specimens. Type specimens allow an objective application of the species name and provide a standard of reference by which the application of that name can be determined [2]. Therefore, this approach offers representative examples of a morphological species concept which allows users a set of objective references when analysing specimens of unknown taxonomic affinity [3]. In practice, one of the principal taxonomic problems faced is the significant level of morphological plasticity exhibited in certain taxonomically important features of the foraminiferal test. This has led to erroneous and inconsistent species identifications, particularly between closely related species where these problematic morphological boundaries are often poorly defined [4, 5].

There are currently very few established quantitative morphological frameworks from which one can consistently identify and assign a specimen into a well-defined species concept [6, 7]. This has led to the prevalent use of an open nomenclature (i.e. ‘lumping’), leading to the potential merging of species based on broad morphological concepts. This is particularly problematic with the assignation of juveniles [8], where their morphologies differ from those of the adult form. The occurrence of numerous polymorphic species incorporating a range of gradational diagnostic features, inevitably leads to erroneous species identification. This in turn introduces error into foraminiferal-based environmental reconstructions (e.g. [9, 10]), some of which underpin the physical science basis for our current understanding of climate change [11].

In order to exploit their impressive and exceptionally long fossil record, it is vital that both extant and fossil foraminifera can be unambiguously attributed to an established and stable taxonomic nomenclature. Only within the confines of such a system can the true taxonomic affinities and biogeochemical, genetic and morphological properties of a valid species be communicated within the academic literature [12, 13]. Palaeoenvironmental research in particular requires a strong taxonomic platform, since the cornerstone of most studies relies on a comparative analysis of modern and fossil species compositions. Our understanding of the ecological niches and biogeographical distributions of modern species can then be applied across time and space [8, 14]. Erroneous species identifications have the potential to undermine the credibility of research, leading to flawed current and future research agendas [15–17]. Such problems lead us to question the degree of stability and reliability in the current, morphology-based species concepts practiced in foraminiferal research. Therefore, it is imperative that a more robust and stable morphology-based taxonomy, rooted in molecular systematics, is developed and adopted.

Over the past 20 years, the focus of taxonomic endeavour has shifted its emphasis away from classical morphology-based taxonomy to concentrate on molecular systematics. Molecular approaches using typically a fragment of the SSU ribosomal RNA gene have enabled the genetic characterisation of single specimens of foraminifera [18]. The extensive genetic data now available highlights the limitations of a taxonomy built purely upon classical descriptions of test morphology. For example, molecular analysis has enabled the delineation of many phylogenetically separate species which were not morphologically discriminated in classical taxonomy i.e. cryptic species (e.g. [5, 19–21]). The potential presence of cryptic diversity has significant implications for the interpretation of palaeoenvironmental records, because faunal analyses which comprise an amalgamation of cryptic genetic types, may compromise the degree of precision in faunal reconstructions [21].

Whilst molecular systematics is widely acknowledged as an important tool for re-examining species level relationships in the living assemblage, it does not provide sufficient evidence alone for its application to the fossil assemblages. Although fossil environmental DNA studies are now possible in deep-sea subsurface sediments [22], fossil specimens cannot be individually characterised using such molecular techniques and can only be practically delineated based on their test morphology. Prior to the development of molecular systematics, the morphological approach to taxonomy in the fossil record, though largely robust, could not resolve many of the practical taxonomic problems faced by the benthic foraminiferal community. Over-reliance upon these singular methods of delineation, be it molecular or morphometric, comes with potentially significant limitations. The tools are now available to combine these different lines of taxonomic evidence to provide an integrated approach to taxonomy.

An integrated foraminiferal taxonomic framework offers the potential to test species boundaries, allowing the development of a framework which can be consistently applied. A recent suite of papers have successfully utilised a combined molecular and morphological approach to delineate between species, in order to revise and redefine many benthic foraminiferal taxonomic positions [5, 20, 23–27]. However, despite considerable technological advancements in imaging techniques over the past 20 years, there has been limited progress in quantitatively delineating species based upon their morphology. Many of the aforementioned studies placed their emphasis on comparing genetic delineations with qualitative morphological descriptions alone. Therefore, these qualitative studies cannot provide repeatable and quantifiable morphometric and genetic analysis conducted on the same specimens. Many of the recent combined taxonomic studies, regardless of the current evidence for taxonomic confusion, continue to attach classical taxonomic names to newly delineated genetic types, nearly always without reference to the original type material. However, this approach carries the inherent danger of reasserting the cumulative taxonomic confusion associated with the historical, sometimes tortuous, synonymy of a morphology-based taxon concept to the newly delineated genetic type.

For application to the fossil record, it is imperative that there is consistency within the nomenclature that is applied to the morphological concepts of foraminifera. In order to connect the present to the past, it is essential that taxonomic delineations based upon molecular systematics are both embedded within the same taxonomic framework based upon morphological systematics. However attractive a solely molecular approach might seem, there is no context from which to effectively communicate these delineations and any attempts to name these genetic types without reference to a morphology-based classification scheme would likely compromise the rules of nomenclature set out by the International Commission on Zoological Nomenclature (ICZN) [2].

This study sets-out a new taxonomic framework from which the recent developments in molecular systematics can be reconciled with traditional morphology-based taxonomy. We aim to test the classical descriptive taxonomic species concept using quantitative morphological measurements and an independent DNA-based component, utilising both museum type specimens and topotypic specimens (i.e. specimen originating from the type locality of the species or subspecies to which it is thought to belong [2]). This study will establish for the first time, a secure methodological approach to benthic foraminiferal taxonomy whereby the formal taxonomic nomenclature of the type material can be mapped onto morphologically characterised topotypic specimens whose contemporary genetic type is established.

**Fig 1:**
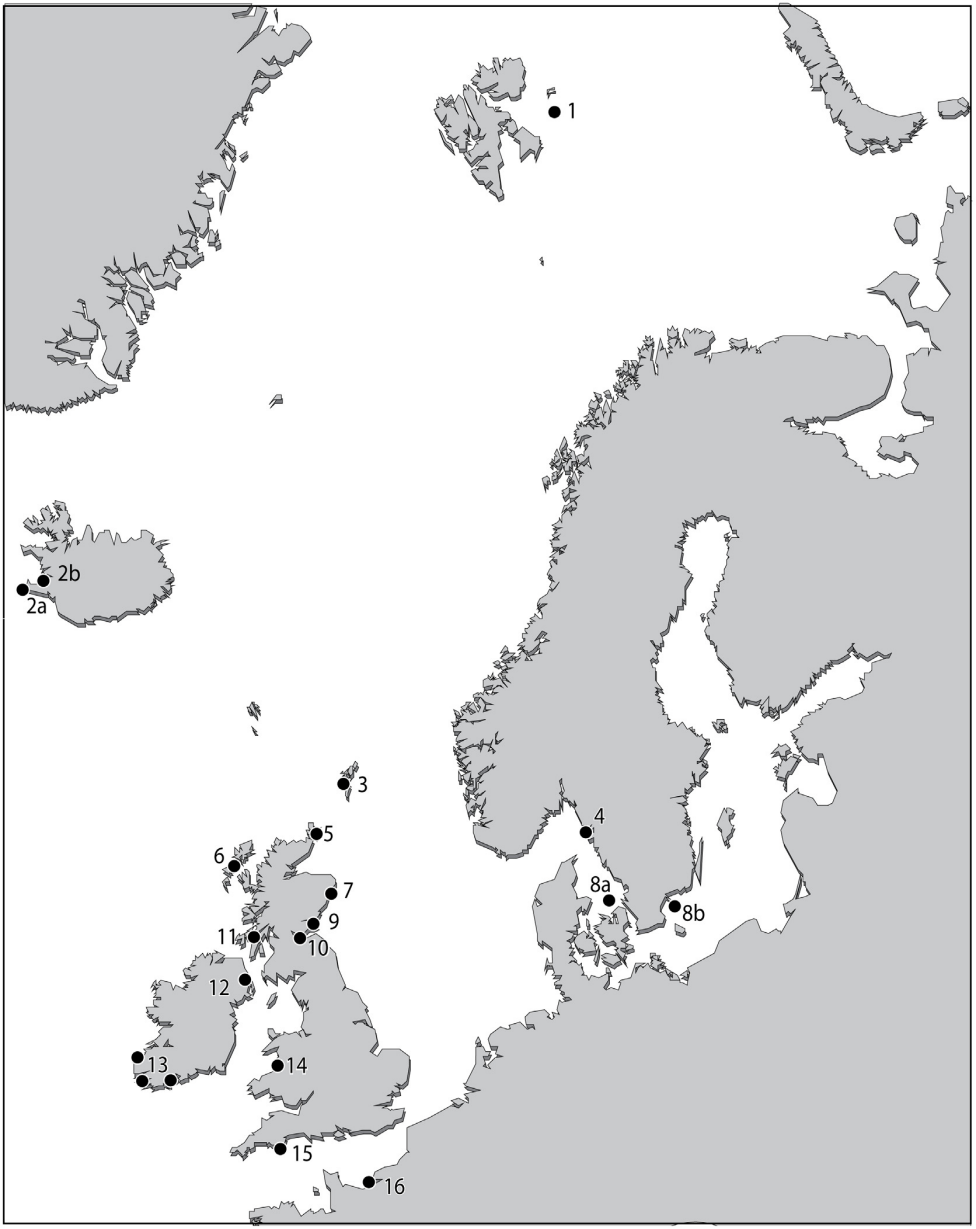
Sample site map of contemporary elphidiid specimens collected across the NE Atlantic. Geographic locations of specimens collected across the NE Atlantic [30] which have been morphometrically analysed in this study. Numbers next to labels correspond to sample sites listed in S1 Table.

In order to achieve these goals, the morphology-based taxonomic concept of *Elphidium williamsoni*, Haynes, 1973 type specimens and descriptions, and *Polystomella umbilicatula* Walker and Jacob, 1798 syntype specimens of Williamson, 1858 were compared with the morphometric and allied molecular identity of contemporary topotype specimens. In addition, we compared the type and topotypic material against the contemporary specimens of the same genetic type collected from 16 NE Atlantic sites (Fig 1). Our aim is to establish whether or not a common molecular signature exists within the morphometric concept of *E*. *williamsoni*. At the same time, this study defines the quantitative morphological boundary of *E*. *williamsoni*, in comparison to other elphidiids which have previously been associated, or even confused with the original *E*. *williamsoni* species concept. The overall aim is to allow for an objective assessment of morphology which can be statistically evaluated to determine if any given specimen, be it fossil or contemporary, conforms to the original morphological concept of *E*. *williamsoni*.

## Materials and Methods

### Material collection

#### Topotype specimens

Contemporary live topotypic specimens were collected from Haynes’ original *E*. *williamsoni* type site location along the Clettwr transect, Dovey Marshes, Wales, (Fig 1, Site 14) [28, 29]. Surface sediment samples (upper 1 cm) were collected by hand with a scraper during a low tide on 28^th^ March, 2013. No specific permissions were required to collect sediments at this site. The sample site is not privately owned or protected in any way and the field study did not involve endangered or protected species.

These samples were processed as follows: specimens were examined under a stereomicroscope and potential living specimens were distinguished by the natural colouration of the protoplasm and were extracted from seawater using a fine paintbrush. These pre-screened specimens were placed in clean seawater and subsequently examined to establish if there was any pseudopodial activity, such as the overnight formation of sediment cocoons around the test or the movement of specimens from a predefined position. Once the live specimens were identified, they were picked, dried and mounted prior to scanning electron microscopy (SEM) imaging. Following SEM imaging, the specimens were individually crushed for DNA extraction and genetic characterisation using an ~1000 base pair fragment of the 3’ terminal region of the small subunit ribosomal RNA gene [30]. In total, 18 topotypic specimens were genetically characterised and their sequences deposited in the Genbank/EMBL database (accession numbers KX228717-KX228734). A further 77 topotypic specimens were SEM imaged only (S1 Table; site location number 14).

#### Type material

**Fig 2:**
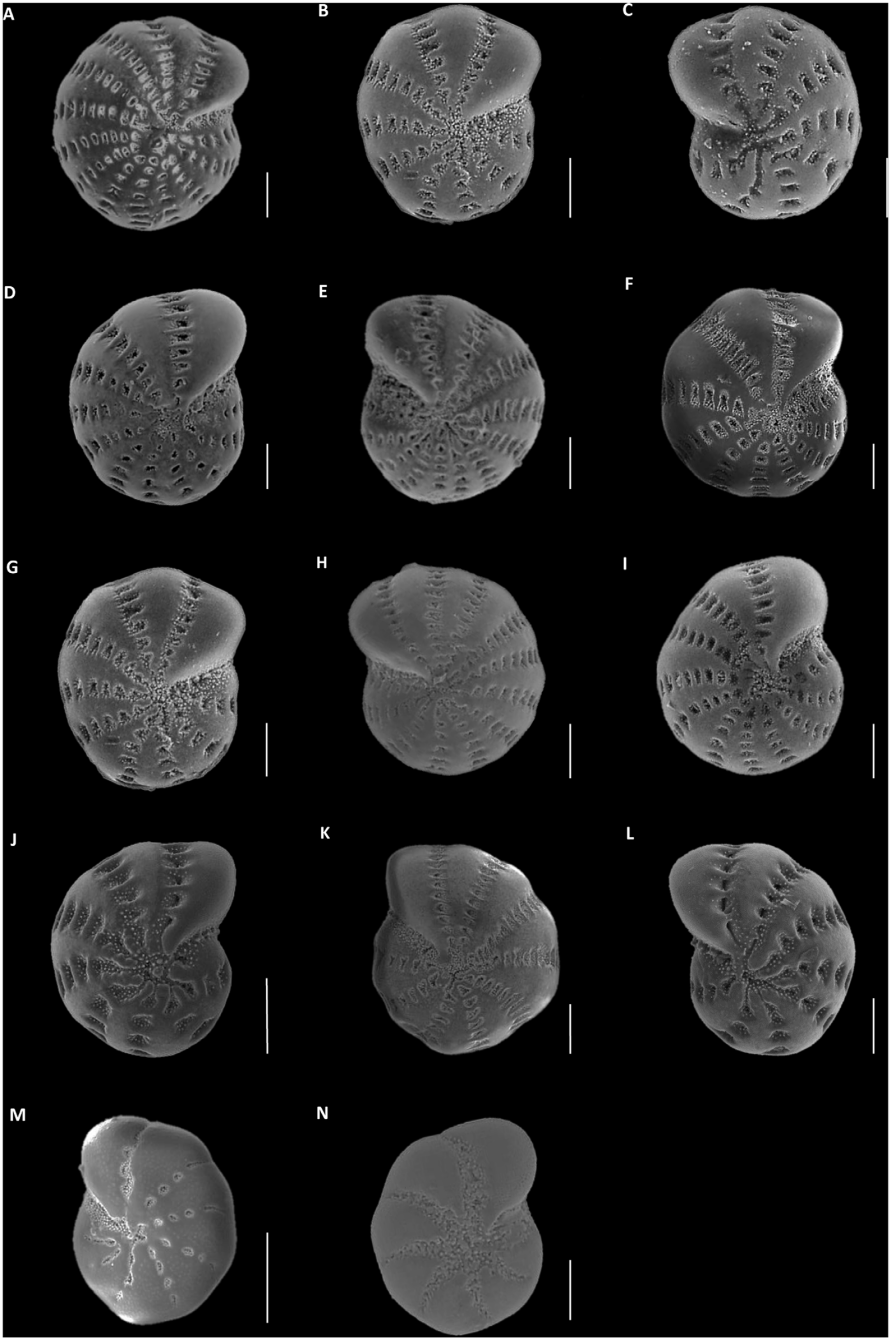
SEM and ESEM images of *Elphidium williamsoni* and elphidiid SSU rRNA genetic types S4 and S5. (A) *Elphidium williamsoni* Haynes, 1973 (holotype specimen), (B-C) *Elphidium williamsoni* (paratypes), (D-F) Contemporary topotypic sequenced specimens, (G-I) *Polystomella umbilicatula* Walker and Jacob, 1798 syntypes of Williamson, 1858, (J-L) elphidiid genetic type S1 specimens collected from across NE Atlantic, (M) elphidiid genetic type S4 [30] and (N) elphidiid genetic type S5 [30]. Scale bars correspond to 100 μm.

The type material was obtained on loan from the Natural History Museum London (NHM) in March 2013. These specimens consisted of *Elphidium williamsoni*, Haynes, 1973 (NHM Reference Number: Slide 1970: II: 26:431–42 (10 paratypes)) and Stub 1970: II: 26:597 holotype), and *Polystomella umbilicatula* Walker and Jacob, 1798 syntypes of Williamson, 1858 (NHM Reference Number: 96.8.13.16 (25 syntypes)). The *Polystomella umbilicatula* syntypes of Williamson, 1858 were included in the analysis, as these represent the first specimens of *E*. *williamsoni* to be described; as noted by Haynes [29], these specimens were erroneously assigned to *P*. *umbilicatula* by Williamson in 1858 [31]. Fig 2 illustrates a selection of type material environmental SEM (ESEM) images that were utilised in this study. These specimens where chosen to portray the range of morphological variability exhibited by the type material (Fig 2, specimens A-C, G-I). These valuable reference materials were unavailable for normal SEM analysis as this would have required gold coating of the specimens. Therefore, specimens were imaged using an ESEM at Herriot Watt University, Edinburgh, UK (April 2013).

### Morphological analysis

#### Quantitative morphological analysis

Morphological analysis was conducted on both the contemporary topotype material and type material. To investigate the morphological similarity between specimens, a combination of 16 morphometric and categorical variables were acquired from the SEM images described above. In order to standardise the method, only SEM side views of the test were used in the analyses (Table 1). The morphological characters measured were derived from and are intended to quantify the key diagnostic features included in the original species description and diagnosis of *Elphidium williamsoni* Haynes, 1973 [29]:

**Table 1:** Test characters measured or assessed and used in morphometric analysis. N = last chamber, N1 = penultimate chamber etc.

Type of character	Name	Variable Number	Method of measurement	Unit/ Category/ Type
Morphometric	Maximum test diameter	1	Maximum diameter of test parallel to axis of coiling	Not included in analysis
Morphometric	Maximum width of the penultimate chamber (N1)	2	Maximum diameter of penultimate chamber calculated from the boundaries of the sutures (i.e. from the end of one suture to the end of the next suture)/maximum test diameter	Ratio
Morphometric	Average septal bar height in the suture between penultimate chamber (N1) and chamber (N2)	3	Average diameter of the first three septal bars (if present) from the umbilical area towards the periphery edge of the foraminifera	Micrometres
Morphometric	Relative difference in the width of the septal bar to the rest of the chamber	4	Difference between the width of septal bar in comparison to rest of the chamber	Ratio
Morphometric	Relative width of sutural furrow in the penultimate chamber (N1)	5	Width of the sutural furrow in the penultimate chamber at the umbilical region/width of the sutural furrow at the periphery of the test	Ratio
Morphometric	Sutural angle along the interseptal space between chambers N1 and N2	6	The curvature of the suture between the final and penultimate chamber	Degree
Morphometric	Total number of chambers	7	Number of chambers visible in the final whorl/maximum test diameter	Ratio
Morphometric	Total number of septal pits	8	Number of complete septal pits (defined and bounded by two septal bars)/maximum test diameter	Ratio
Morphometric	Roundness of the foraminiferal test	9	As calculated from the outline of the entire shape: 4*area/(π*major_axis^2)	0–1
Morphometric	Average roundness of the septal pit	10	Mean roundness of the pit averaged across the foraminifera, as calculated from the outlines of the septal pits: 4*area/(π*major_axis^2)	0–1
Morphometric	Relative proportion of septal pit area to rest of the test	11	The relative proportion is calculated by total foraminiferal area/total area of the septal pits	Ratio
Morphometric	Maximum width of umbilical bosses	12	Maximum width of umbilical bosses in the umbilical area/maximum test diameter	Ratio
Categorical	Porosity (Strength)	13	Strength of width of pores. The average of the ten closest pores were measured from the junction of chambers N1 and N2. The average width of these pores was then calculated and this average was grouped into one of three categories: fine pores <1 μm, medium pores 1–2 μm or large pores > 2 μm	Fine-1
				Medium-2
				Coarse- 3
Categorical	Degree of apertural ornamentation	14	Angularity of tubercles around the aperture	None-1
				Very weak-2
				Weak-3
				Medium—4
				Strong-5
Categorical	Degree of openness of the umbilical area	15	Degree of openness of the umbilical area	None-1
				Very weak-2
				Weak-3
				Medium—4
				Strong-5
Categorical	Degree of ornamentation within the sutures (including pits)	16	Angularity and regularity of tubercules within sutures	None-1
				Very weak-2
				Weak-3
				Medium—4
				Strong-5

**Diagnosis**: “A rotund species of *Elphidium* with rounded periphery and slight, rather flat umbilicus on each side filled with irregular ends of the chambers. Fossettes and septal bars well developed, reaching about eight or nine in number on each side and covering about half of the chambers. Up to 14 chambers visible. Wall smooth with relatively sparse tubercules within the septal pits and at the base of the apertural face.”

**Description**: “Test semi-inflated, slightly umbilicate with rounded periphery, entire becoming semi-lobate at the last few chambers- chambers arranged in an involute planispire, 13 visible, slowly increasing in size with marked septal pits (fossettes) increasing from six to eight or nine on each side (ten on third chamber from the last), strong, narrow septal bars almost equal in length to rest of each chamber, in one case (on the last chamber) with a proximal opening, pits lozenge shaped, tuberculate within; septal sutures flush- not visible; wall radial, finely perforate, pores less than 1 micron in diameter, tuberculate below the apertural face; aperture a series of irregular openings along the basal suture of the last chamber, linking with pits of the first exposed chamber”.

A combination of Image Pro Express and ImageJ 1.47 software [32] were used to collect the morphometric measurements. To calculate foraminiferal test roundness and area (Table 1), a trace measurement tool was used in the Image Pro express software, which created a line feature around the periphery of the foraminiferal test. This line feature was imported into ImageJ and an automatic thresholding procedure was then applied to determine the foraminiferal test area. Additionally, in order to quantify the morphological traits of the septal pits, each SEM image was imported into Adobe Illustrator CS6 software and the septal pits were manually digitised using a graphics tablet. These septal pit outlines were imported into the ImageJ software and an automatic threshold procedure was then applied to the septal pit outlines and the ‘analyse particles’ tool was used to calculate the morphometric measurements. Calibration for the foraminiferal test and septal pit measurements were calculated using the known length of the scale bar of the SEM images in micrometres.

Infilling procedures following the methodology of Hayward et al. [20] were utilised when morphological characters were obscured by debris or when the test was broken. This accounted for 0.21% of the total features measured. The morphological matrix was standardised by ranging the variation between each character from 0 to 1, following the methods of Hayward et al. [20].

#### Morphological distinctiveness and interspecific variability

For the purpose of investigating the morphological distinctiveness and interspecific variability, two morphologically similar yet genetically distinct outlier groups (elphidiid genetic types S4 and S5 from Darling et al.[30]) were utilised in the morphological analysis. These outlier groups were chosen because, based on traditional taxonomic concepts, their morphological characteristics have previously been confused, resulting in their designation as members of the *Elphidium excavatum* (Terquem) complex. To add to confusion, the morphospecies concept of *E*. *williamsoni* has been previously named *E*. *excavatum* [28, 33–41] and *E*. *williamsoni* has also been previously considered a subspecies of the *E*. *excavatum* complex, under the name of *E*. *excavatum williamsoni* [42, 43]. Darling et al.[30] used the distinct morphological profiles of the genetic types S4 and S5 test SEM images as the basis for the taxonomic designations of S4 and S5 as *E*. *clavatum* and *E*. *selseyense* respectively.

In order to compare the potential range of morphological variation in *E*. *williamsoni* captured by the museum type material and the contemporary topotype specimens with the variation present across its biogeographic range, we morphologically examined a further 213 specimens of the same genetic type collected from the NE Atlantic shelves by Darling et al. [30] (Fig 1). No specific permissions were required for the collection of these samples. The sampled locations are not privately-owned or protected in any way, and the field studies did not involve endangered or protected species.

### Data analysis

The morphological data was analysed using a principal coordinate analysis (PCO), an unweighted pair group method with arithmetic mean (UPGMA) cluster analysis, and a discriminant function analysis (DFA). These statistical tests were performed using a combination of PAST v.13 [44], dendroUPGMA [45], and SPSS v.22 software. To reduce the dimensionality of the dataset, a PCO was performed upon all the morphometric characters collated from the contemporary topotypic material and the NHM type series collections in PAST software. In addition, a second PCO analysis was performed, whereby the additional 213 genotyped specimens from the NE Atlantic were added into the analysis.

A UPGMA cluster analysis was used to generate a cluster diagram of the morphological relationships between the topotypic material, NHM type material, the additional NE Atlantic specimens of the same genetic type and the genetically distinct elphidiid outliers (genetic types S4 and S5).

Finally, a DFA was calculated from the results of the standardised dataset to establish the key diagnostic criteria which can be used to reconcile molecules and classical type concepts in order to aid classification of specimens into each genetically distinct group. The robustness of the assignment is assessed through a resampling cross-validation procedure in SPSS v.22.

## Results

### Genetic characterisation

Using the partial SSU rDNA 3' end region, all the contemporary topotypic specimens collected from the Dovey Marshes were genetically characterised as the distinct elphidiid genetic type S1 (GenBank accession numbers KX228717-KX228734), which has been widely identified across nine biogeographic zones in the NE Atlantic [30]. Sequences of this genetic type have previously been deposited in GenBank [46] (consulted in November 2015) by Langer [47], Ertan et al. [48], Pillet et al. [49], Grimm et al. [50], Pillet et al. [27], Habura et al. [51] and Camancho et al. [52].

### Morphological analysis

#### Morphological differentiation between type and topotypic material

**Fig 3:**
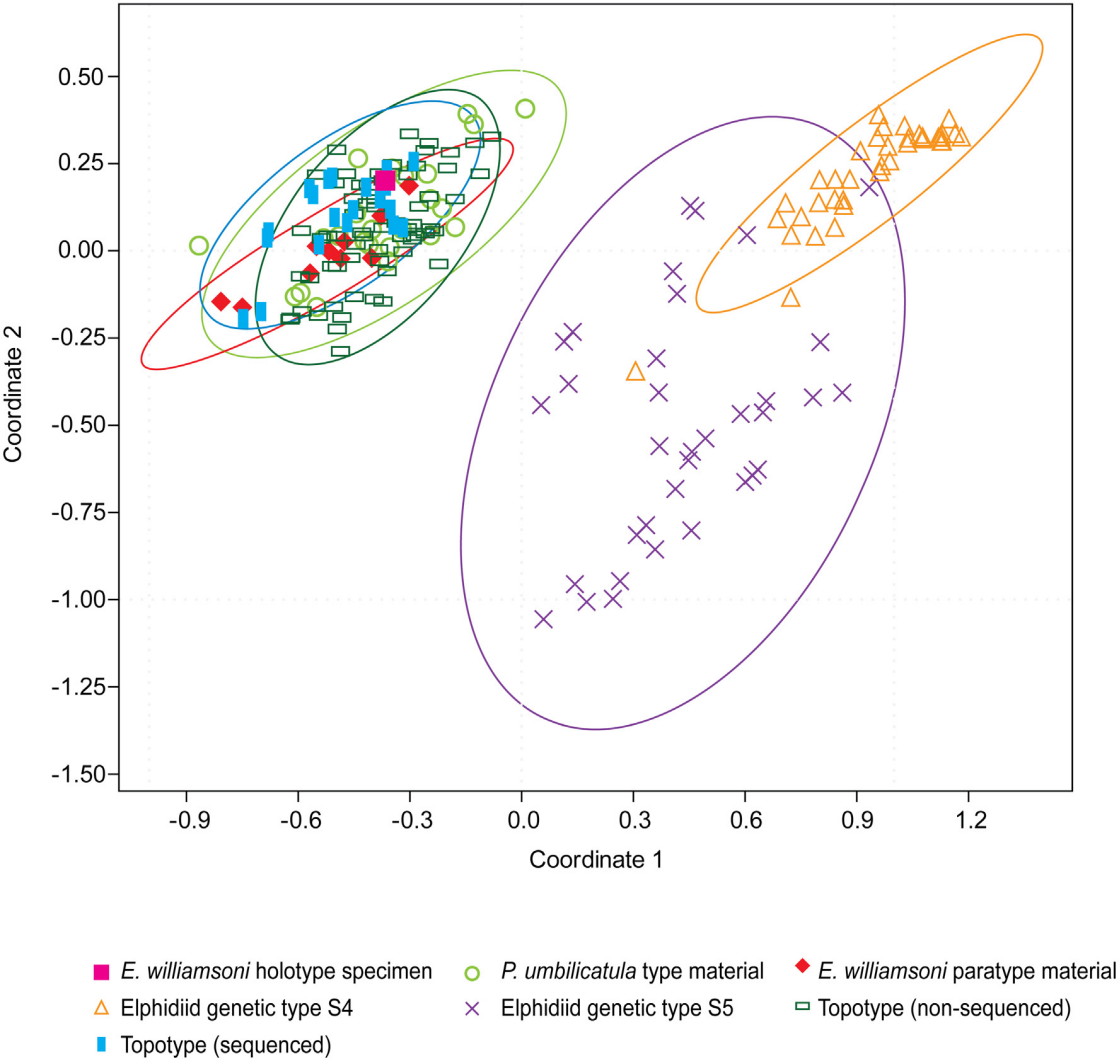
Principal coordinate analysis. Bi-plot of the PCO analysis based on the morphological characters of *Elphidium williamsoni* Haynes, 1973 type specimens, contemporary topotypic specimens, *Polystomella umbilicatula* Walker and Jacob, 1798 syntypes of Williamson, 1858 and the two outlier elphidiid genetic types S4 and S5. These groups are bounded by 95% confidence ellipses. The first two principal coordinates account for 59.9% of the total variance.

**Fig 4:**
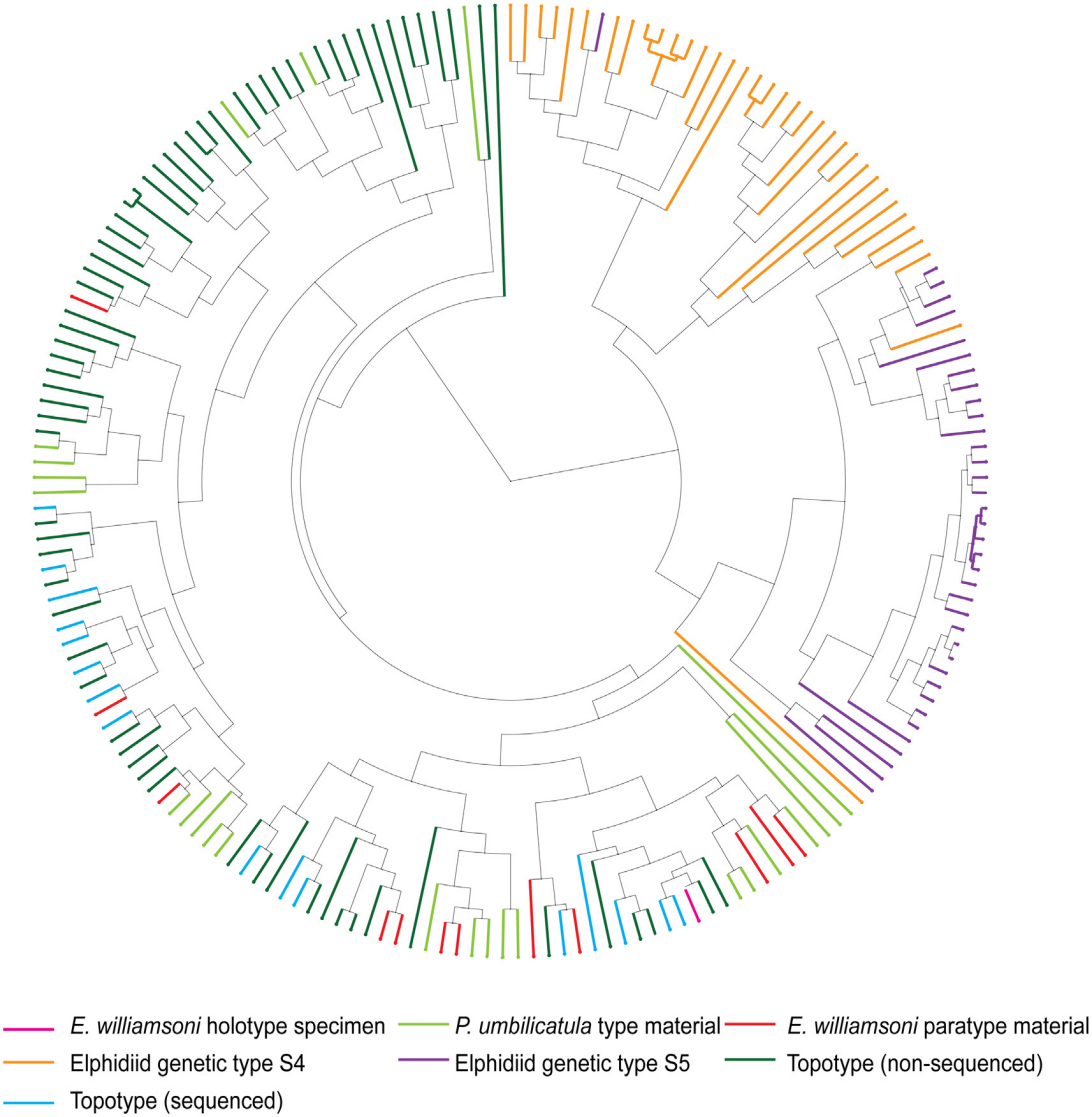
UPGMA cluster analysis dendrogram. UPGMA cluster analysis tree based on the morphological characters of *Elphidium williamsoni*, Haynes, 1973, contemporary topotypic specimens, *Polystomella umbilicatula* Walker and Jacob, 1798 syntypes of Williamson, 1858 and the two outlier elphidiid genetic types S4 and S5.

The results from the UPGMA cluster analysis and the PCO analysis (Figs 3 and 4) illustrate that Haynes’ original type description and species concept can be reconciled with the contemporary topotypic material.

A PCO of the assessed morphological characters was utilised to determine the relationship between the morphology of the topotypic material from Aberdovey, Wales and the morphology of the type material from the NHM (Fig 3). The results of the PCO indicate that there is morphological congruence between the type and topotypic material. Most of the variation common to all of these forms is described by the first two principal coordinates (PC) which account for 59.9% of the total variance. The results illustrate that there are three morphologically distinct clusters of specimens and that the type and topotypic material are strongly segregated from the genetically distinct elphidiid S4 and S5 outlier specimens. Moreover, it can be demonstrated from the 95% confidence ellipses that Haynes’ 1973 type material, including the holotype, is situated within the centre of the morphospace occupied by the contemporary topotypic specimens sampled in 2013. However, it should be noted that there is some morphological overlap between the genetic outlier groups as evidenced by the 95% confidence ellipses; notably there are four outlier specimens (two genetic type S4 and two genetic type S5) which do not cluster with the majority of their respective genetic types within the PCO morphospace (Fig 3).

The results from the UPGMA cluster analysis (Fig 4) confirm the results from the PCO analysis, that the type and contemporary topotypic specimens are morphologically distinct from the genetic outliers. Overall, the UPGMA cluster analysis highlights that three main morphological groups can be determined, despite some morphological overlap between six specimens from the two genetically distinct outlier groups (Fig 4).

#### Multivariate analysis between topotype, type material and *Elphidium* genetic type S1 specimens across the North East Atlantic

In order to determine whether the full extent of morphological variability of *E*. *williamsoni* has been captured from the type material of Haynes, 1973, the morphological attributes of the topotypic and type material were compared and analysed against the morphology of 213 *Elphidium* genetic type S1 specimens [30], collected from across the NE Atlantic (Fig 1, S1 Table).

**Fig 5:**
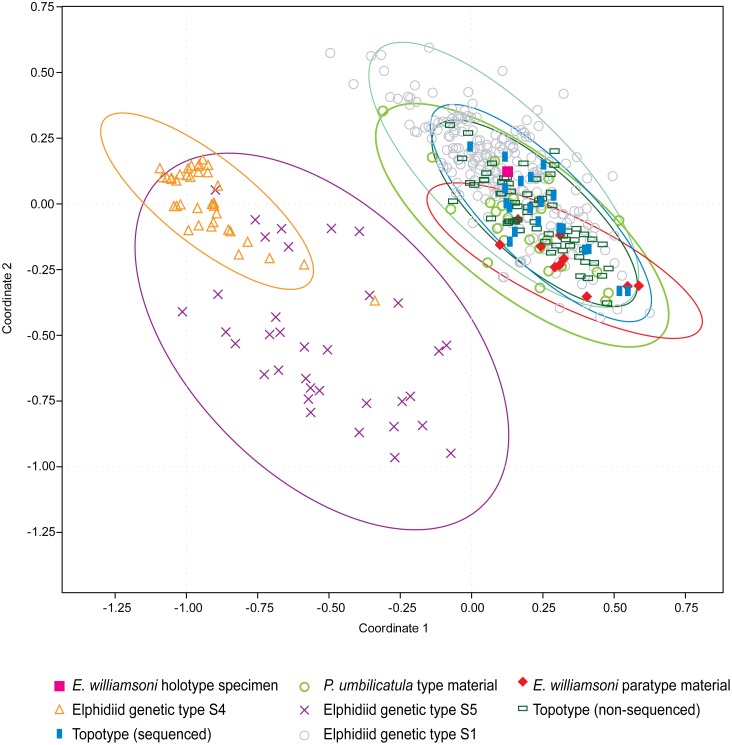
Principal coordinate analysis. Bi-plot of the PCO analysis based on the morphological characters of *E*. *williamsoni* Haynes, 1973 type specimens, contemporary topotypic specimens, *Polystomella umbilicatula* Walker and Jacob, 1798 syntypes of Williamson, 1858, contemporary elphidiid genetic type S1 specimens and the two outlier elphidiid genetic types, S4 and S5. These groups are bounded by 95% confidence ellipses. The first two principal coordinates account for 48.5% of the total variance.

Haynes’ 1973 type specimens of *E*. *williamsoni* fall within the morphological variability of all the *Elphidium* genetic type S1 material collected from across the NE Atlantic, as illustrated from the 95% confidence ellipses (Fig 5). The results from the PCO analysis indicate that the first two PCs describe 48.5% of the total variance. The results indicate that the genetically distinct outlier groups clearly separate themselves from the type and topotypic material. However, using these characteristics some morphological overlap is also observed between the genetically distinct outlier groups.

**Fig 6:**
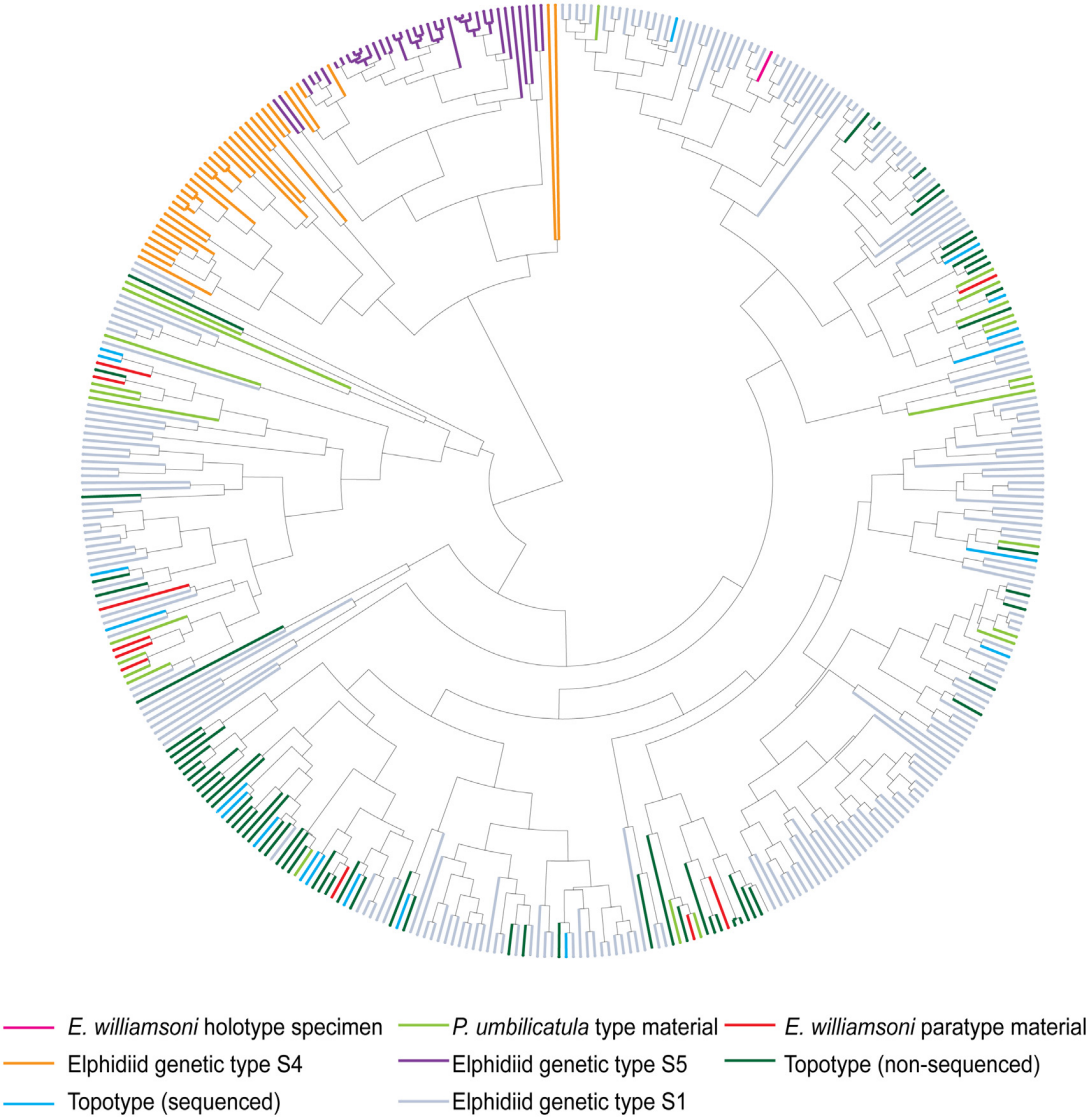
UPGMA Cluster analysis dendrogram. UPGMA cluster analysis tree based on the morphological characters of *Elphidium williamsoni*, Haynes, 1973 type specimens, *Polystomella umbilicatula* Walker and Jacob, 1798 syntypes of Williamson, 1858, contemporary elphidiid genetic type S1 specimens collected from across the NE Atlantic shelves and the two outlier elphidiid genetic types S4 and S5.

The results from the UPGMA cluster analysis (Fig 6) highlight that three genetically distinct forms (corresponding to genetic types S1, S4 and S5) can be separated based upon their morphology. Fig 6 indicates that the topotypic specimens are situated across multiple clusters, suggesting that this material has captured a significant proportion of the morphological variability exhibited by *E*. *williamsoni* from across the NE Atlantic. Again, it is also evident that some morphological overlap can be observed amongst the genetic outlier specimens S4 and S5 based on the morphological characteristics employed.

#### Morphological discrimination of *Elphidium williamsoni*

A DFA was performed on the dataset to identify key characters in order to aid classification of specimens into the genetically and morphologically assigned concept of *E*. *williamsoni*. In order to optimise the morphological interspecific discrimination of *E*. *williamsoni*, the DFA was performed utilising the genetically defined groups as *a priori* groupings (type material was combined with the genotyped topotypic material based on the results of the PCO and cluster analyses, Figs 5 and 6 respectively). The DFA showed good membership within the three genetic types, and the percentage of the total overall correct classification of specimens accurately assigned into the genetic groups is 98.1% and 97.6% after the cross validation procedure (Wilks: 0.20, p: <0.05). The results illustrate that the specimens within elphidiid genetic type S1 (including the type and topotypic material) are distinct morphological entities from the genetic outlier groups S4 and S5 (Table 2). The observed cases of misclassification in the DFA and cross validation analysis only occur between the two genetic outlier groups (Table 2).

**Table 2:** Percentage of correctly allocated specimens by the DFA.

			DFA confusion matrix		
Genetic type	Percentage of specimens correctly classified in the DFA	Percentage of specimens correctly classified in cross validation analysis	Genetic type S1	Genetic type S4	Genetic type S5
**Genetic type S1**	100	100	347	-	-
**Genetic type S4**	71.1	71.4	-	27 (25)	8 (10)
**Genetic type S5**	100	100	-	-	37

Percentage of specimens correctly classified into their respective genetic type based on their morphological characteristics in the DFA and cross validation analysis. The confusion matrix of the number of specimens correctly and incorrectly classified by the DFA and cross validation procedures is also illustrated. The numbers shown in brackets depict the differences in the classification assignments in the cross validation analysis.

Overall, the results of the DFA indicate that each genetic type exhibits discrete interspecific diagnostic morphological characters. The key morphological characters which delineate these genetically distinct species include (with reference to Table 1): porosity (13), total number of septal pits (8), openness of umbilical area (15), ratio of septal pit area to the rest of the chamber (11), septal pit roundness (10) and average ratio of the septal bar width to the rest of the chamber (4). These identified quantitative morphological boundaries can be utilised in the future as a foundation for the morphological recognition of *E*. *williamsoni*.

## Discussion

**Fig 7:**
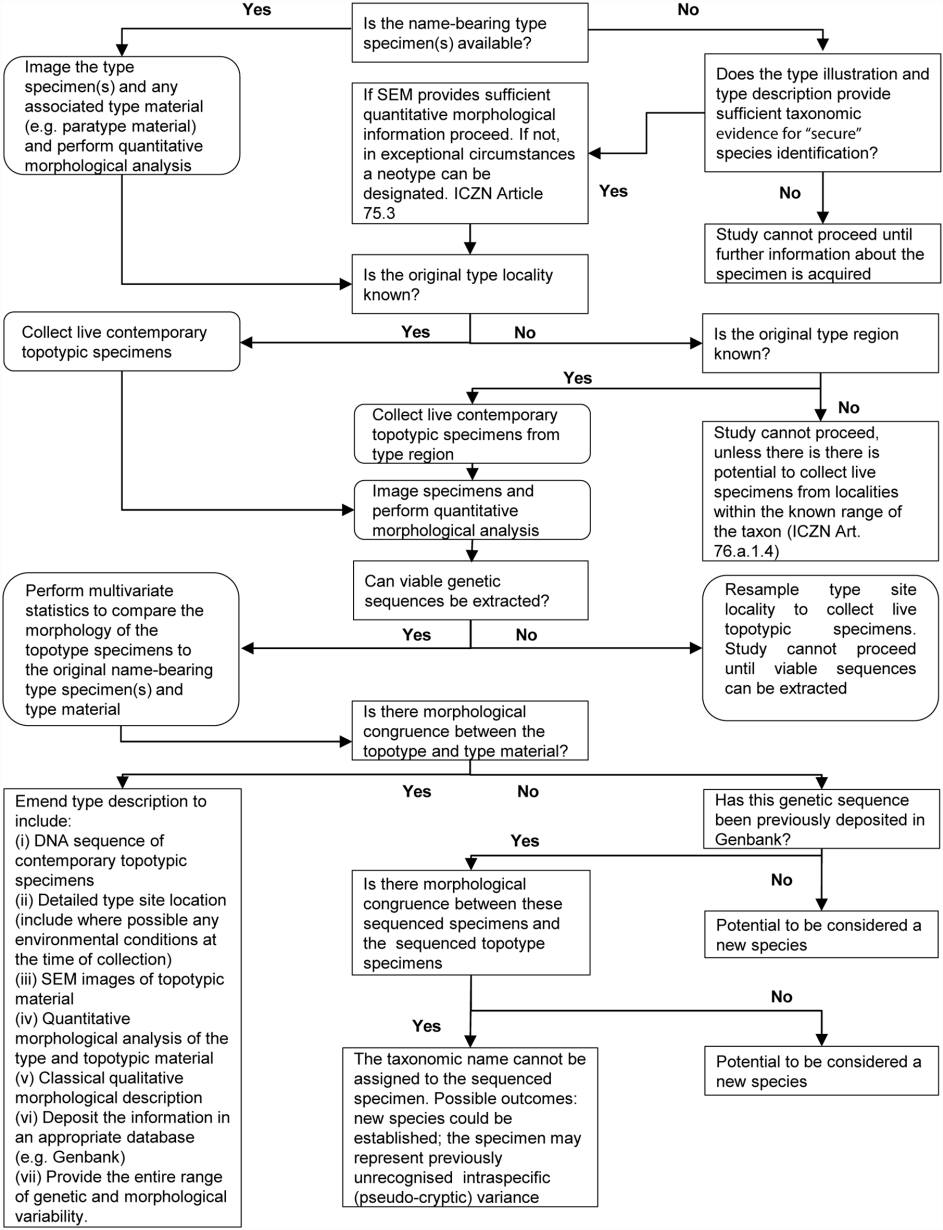
Taxonomic framework for reconciling molecules and morphology. The initial protocol requires (i) a candidate specimen with distinctive test morphology, (ii) the potential for DNA extraction and (iii) a comprehensive and detailed literature review, to allow a qualitative morphological comparison of the candidate specimen against the type descriptions and illustrations.

This study provides a new taxonomic framework (outlined in Fig 7) that integrates partial SSU rRNA gene sequences of contemporary topotypic specimens and quantitative morphometric analysis of type and contemporary topotypic material, to reconcile the morphological species concept to a distinct genetic type. This study utilises Haynes’ 1973 *Elphidium williamsoni* type material to implement this framework.

### *Elphidium williamsoni* and the *Elphidium excavatum* complex

*Elphidium williamsoni* was chosen as the first benthic foraminiferal taxon for applying this integrated analytical approach for several reasons. It is used extensively in palaeoenvironmental studies [53, 54], particularly in proxy-based relative sea level (RSL) reconstructions due to its strong and quantifiable relationship within inter-tidal zones. Understanding the true intraspecific morphological variation within *E*. *williamsoni* would enable comparative high-resolution environmental studies to be carried out throughout its biogeographic range. This has only recently become possible due to the large number of genotyped specimens with corresponding SEM images (n = 213) generated during an extensive biogeographical study in the North East Atlantic [30], which have now become available for morphometric analysis. To complement this, Haynes’ original type material of *E*. *williamsoni* was available for analysis from the NHM London and Haynes’ original type site location was also recorded in detail and could be easily accessed [29].

Resolving the taxonomic identity of *E*. *williamsoni* has always proved challenging because this morphospecies is a member of one of the largest and most morphologically diverse groups of benthic foraminifera. Delineating species within the *Elphidium* genus and elphidiids in general has posed a significant challenge to taxonomists due to the considerable amounts of intraspecific and interspecific variation exhibited in the key morphological characteristics [4]. Thus, considerable taxonomic uncertainty has been conferred upon the species and as a consequence, its species concept has been subject to continued emendation. *Elphidium williamsoni* was originally collected in 1858 by Williamson [31] who incorrectly assigned specimens into *Polystomella umbilicatula*, Walker and Jacob, 1798 [55]. This morphospecies was then later reclassified into the genus *Elphidium* and was renamed *E*. *williamsoni* in Williamson’s honour by Haynes in 1973. However, this species also has phenotypic similarities with other elphidiid species, which has led to it being confused with *Cribrononion* cf *alvarezianum* [56], *Polystomella striatopunctata* [57], *Elphidium umbilicatulum*, [31] and *Elphidium articulatum* [58].

*Elphidium williamsoni* has also been considered to belong to the *Elphidium excavatum* complex, and has consequently been previously named *E*. *excavatum* [28, 33–41]. Furthermore, it has been considered a possible subspecies of *E*. *excavatum* complex, under the name *E*. *excavatum williamsoni* [42, 43]. The two genetically distinct elphidiid outliers (genetic types S4 and S5) used for comparative morphological analysis in this study have also been designated as members of the *E*. *excavatum* complex. They were therefore specifically selected for inclusion within the analysis to help unravel the morphological confusion associated with the *E*. *excavatum* complex. All three complex members are genetically highly distinct from one another [27, 30], yet morphologically quite similar. Darling et al. [30] used the morphological profiles of the genetic types S4 and S5 test SEM images as the basis for the taxonomic designations of S4 and S5 as *E*. *clavatum* and *E*. *selseyense* respectively (see methods). However, in order to fully clarify the taxonomic position of the two genetic outlier groups, further work should be undertaken to reconcile these genetic types with type material adhering to the framework outlined in Fig 7. Nevertheless, performing this additional analysis is considered beyond the scope of this present study.

### Morphometric analysis

The integrated taxonomic, genetic and morphometric framework adopted here has enabled us to verify the robustness of Haynes’ 1973 original taxonomic description and type material of *E*. *williamsoni* against the contemporary topotypic material. We demonstrate that there is strong morphological congruence between the *E*. *williamsoni* type specimens and contemporary topotypic material, as they distinctly group together. Therefore, our results presented here strongly support the conclusions of Pillet et al. [27] that *E*. *williamsoni* is a genetically distinct species, and consequently should not be considered as a subspecies of the *E*. *excavatum* complex.

It is important to note that whilst the genetic outlier specimens (elphidiid genetic types S4 and S5) are always morphologically distinct from the *E*. *williamsoni* genetic type S1, a few specimens of the S4 and S5 outlier specimens do not always cluster within their respective genetic type (Figs 5 and 6). This is due to the limitations in the range of key diagnostic features chosen for analysis, which were specifically derived from Haynes’ 1973 type description of *E*. *williamsoni*. The morphological overlap between these two genetic outlier groups resolves when additional diagnostically important morphological characters related to the genetic outliers (e.g. imperforate collar around the umbilical area) are added to the analysis. Therefore, it is important to highlight that within any morphometric study, the key morphological character combinations that help to delineate species will change with the choice of genetic outlier.

Nevertheless, the morphometric characters used in this analysis are optimal for determining the morphological congruence between the type and contemporary topotypic material. The results illustrate that the morphological characters of Haynes’ 1973 type specimens have captured a significant proportion of the intraspecific morphological variation, as these specimens fall within the morphological range exhibited by the contemporary topotypic material (Fig 3). However, it is also important to acknowledge that these specimens do not encompass the entire breadth of intraspecific morphological variability within this species (Figs 5 and 6). This is unsurprising, as there are only 11 type specimens available for the comparative analysis, thus these specimens are unlikely to represent the entirety of intraspecific morphological variation exhibited by *E*. *williamsoni* throughout its biogeographic range. An example of the variability of the morphological characters exhibited by the contemporary specimens is illustrated in Fig 2 (specimens J-L). This data set, consisting of 213 morphometrically analysed tests of genetic type S1 collected from across the NE Atlantic (S2 Table) has the potential in the future to be used for investigating morphological variation within different populations of *E*. *williamsoni* across distinct biogeographic zones.

### Key diagnostic features of *Elphidium williamsoni* following morphometric analysis

Haynes’ 1973 [29] type description of *E*. *williamsoni* emphasises certain key diagnostic morphological test features to aid future identification of this taxon. The results from the DFA illustrate that many of these key diagnostic features (e.g. openness of the umbilical area, total number of septal pits, porosity, ratio of septal pit area to rest of the chamber and septal pit roundness) are important test features in determining interspecific relationships between the *E*. *williamsoni* type specimens and the genetically distinct outliers. The important diagnostic features highlighted in this study correspond to Haynes’ original description and diagnosis.

However, our results also demonstrate that other diagnostically important features recognised by Haynes, such as septal pit ornamentation (tubercules), test peripheral roundness and total number of chambers, were not as significant in our comparative analysis of *E*. *williamsoni* against the S4 and S5 outlier groups. Nonetheless, in the future these seemingly less important characteristics could become fundamental in determining interspecific relationships against other elphidiid species or may become crucial for improving our understanding of intraspecific variation (due to ontogeny or environmental conditions). Our results highlight that there is not a single morphological character which can be used to delineate the genetic types; instead a combination of morphological characteristics are required for successful discrimination. This conclusion not only supports the value of Haynes’ original type description and diagnosis, but also attests to his taxonomic skill in choosing type material which is representative of morphological variability within the species concept of *E*. *williamsoni* (Fig 5).

### Taxonomic challenges to a fully integrated approach

Previous studies of benthic foraminifera have encountered significant difficulties in reconciling classical taxonomic names to genetic types [5, 20]. Taxonomic challenges may be encountered when implementing a fully integrated approach as resampling of contemporary live topotypic material from the original type locations may be problematic, especially if the type specimens were collected hundreds of years ago [5]. Additionally, the biogeographic distribution of the type species in question may have changed over time due to varying environmental conditions [59]. This is especially important if the original type specimens were collected at the edge of their biogeographic range, as it may be more vulnerable to changes in environmental conditions since the original sampling. Range migration is now becoming more common, as taxa respond to our fast changing climate [59]. Furthermore, there is also the potential for new species to occupy the type-site after the type specimens were collected. These scenarios reinforce the importance of a quantitative comparison of the original type series material against genetically characterised contemporary topotypic material.

Another potential challenge faced when implementing the taxonomic framework includes the possible misplacement or loss of the original type material [3]. The ICZN, Article 73.14 states the absence of a type specimen does not always invalidate the designation [2]. However, many of the original type specimens have been poorly depicted with simplistic line illustrations that often neglect many of the important key morphological features [60]. Therefore, in exceptional circumstances where the original material is lost or the type illustrations and SEM images provide insufficient detail for robust species delineation, a neotype can be designated. The designation of this neotype should follow the requirements set out by ICZN Article 75 [2].

Caution should be exercised when determining and designating a new species. It is crucial to establish if the candidate specimen has any morphological and/or genetic similarity to previously described type material, type descriptions or illustrations. Once genetic distinctiveness is established, it is vital to closely examine the morphological identity, particularly when a new genetic type had not been morphologically discriminated prior to genetic characterisation. In addition, it is important to note that a candidate specimen may not always have morphological congruence to type material. Whilst name bearing type specimens are vital reference points for the assignment of a taxonomic name, these specimens are typically chosen in order to portray the exaggerated morphological features of the species in question [3, 61]. As a direct consequence, in an applied taxonomic situation a user often only has a few catalogued morphological end members from which they can choose and apply a taxonomic name and species concept to a specimen. There is therefore the potential to encounter a greater degree of morphological variation within a genetically distinct species which has not been encapsulated by the type material (as shown in Fig 5). Thus, there should be a concerted effort to sample, analyse, archive, image and quantify the entire range of morphological and genetic variability exhibited by a species, so that in the future the process of designating a new species is more transparent and robust.

The proposed taxonomic framework in this study (Fig 7) consolidates the progressive integrated benthic foraminiferal taxonomic studies such as those provided by Holzmann [5], Hayward et al. [20], Tsuchiya et al. [25], Pillet et al. [27], Schweizer et al. [62, 63], Darling et al. [30] and Roberts [64]. We hope these approaches will therefore reduce the over-reliance upon the individual taxonomist’s judgement for species delineation of other extant and fossil foraminifera in the future.

### Scientific communication of species concepts

The fusion of the morphometric and molecular taxonomic evidence provided through the proposed taxonomic framework implemented in this study (Fig 7) is only useful if there is a taxonomic setting from which we can communicate these delineations within the academic literature. Traditionally, the distribution of taxonomic knowledge within the academic community has tended to compound the complexity of foraminiferal taxonomy [60]. Some of this confusion can be associated with the fact that few studies provide accompanying SEM or light microscope images. In addition, there is widespread use of different terminologies and morphological characters used to describe and define a species. This raises the question of how can a reliable comparative assessment of taxa occur across time and space, and how can one implement the proposed taxonomic protocol to reduce this confusion in the future?

The development of the new, less expensive imaging techniques and the formation of digital online databases such as the molecular databases GenBank [46] and foramBARCODING [65] and taxonomic databases such as the World Foraminiferal Database [66], and www.foraminifera.eu [67] offer the potential for open access communication of taxonomic knowledge. In particular, they provide a platform to distribute and debate images associated with taxonomic names. However, whilst these resources are becoming more and more valuable for applied taxonomic studies, a consistent approach to taxonomy is required. This is of particular importance because workers producing independent genetic data sometimes continue to reattach a taxonomic name to a genetic type without returning to the original type material and species description. Thus, it should be emphasised that these online databases should complement and not replace the curation of original type material. Nevertheless, these online databases provide a platform from which new species constructs can be developed and openly shared, while also allowing traditional species constructs to be critiqued.

## Conclusions

Future taxonomic studies should focus on the integration of multiple lines of taxonomic evidence to delineate species. Each foraminiferal taxonomic species description should ideally include: (i) a genetic sequence, (ii) detailed quantitative morphological measurements, (iii) traditional morphological descriptions, (iv) detailed type locality information, (v) SEM image of the holotype specimen and should follow the taxonomic and nomenclatural guidelines set-out by ICZN [2]. It is imperative that empirical evidence of the full range of morphological and genetic variability is reliably recorded within the type descriptions, as a longstanding major problem of morphospecies identification has been the inadequate quantification of the whole range of variability in the original descriptions of almost all foraminiferal species so far named. A more objective approach would be to have a representative series of type specimens which encapsulate the range of morphological variability within a population or across different biogeographical ranges. It is also essential that type material is properly archived for future reference, for example through its deposition in a national museum. These recommendations apply equally to the erection of new fossil species except that of course (i) cannot be applied.

This case study of *Elphidium williamsoni* highlights the importance of an integrated taxonomic approach to resolving the taxonomic complexity faced by the benthic foraminiferal community today. Since Williamson’s first description in 1858 of *E*. *williamsoni*, which he incorrectly assigned to *P*. *umbilcatula* (itself first named by Walker and Jacob in 1798 [55]), this study now presents the first clear link between morphologically characterised type material (to which the formal name *E*. *williamsoni* is directly attributable) and the unique genetic type of *E*. *williamsoni*. The taxonomic framework proposed here provides a bridge between molecular and morphological evidence, and its implementation could provide increased rigour for species identification and discovery. It also has the potential to be robust enough for new character definitions, new species and new lines of taxonomic evidence to be added in the future. If other key taxa are systematically redefined, this would provide a foundation for a transformational change to benthic foraminiferal taxonomy.

## Supporting Information

S1 Table Sampling localities and information of individual specimens analysed in this study. (DOCX)

S2 Table Morphological parameters extracted from SEM images used in the analysis. (XLSX)
