# Supplementary material for: A New Integrated Approach to Taxonomy: The Fusion of Molecular and Morphological Systematics with Type Material in Benthic Foraminifera
Source: PLoS One. 2016 Jul 7;11(7):e0158754. doi: 10.1371/journal.pone.0158754 (PMC4936703; doi:10.1371/journal.pone.0158754)
Supplement: S1 Table — (DOCX) [file pone.0158754.s001.docx]

**Table S1. Sampling localities and information of individual specimens analysed in this study.**

| **Site Location Number (Fig 1)** | **Location** | **Sub-sampling site** | **Co-ordinates** | **Number of specimens morphologically analysed** |
| --- | --- | --- | --- | --- |
| 1 | Svalbard (Sv) | SV11-HH11-16A-BC | 79° 41’ 15.06”N 34’ 04.62”E | S4:2 |
| 2 | Iceland (Is) | 2a) Is10-Osar1, Reykjanes Peninsula | 63° 56' 28.00”N 22° 38'55.00”W | S1:2 |
|  |  | 2b) Is10-Geldinganes, Reykjanes Peninsula | 64° 09 '31.00”N 21° 47' 15.00”W | S1:2 |
|  |  | 2b) Is10-Grafarvogur, Reykjanes Peninsula | 64° 07' 57.00”N 21° 48' 23.00”W | S1:4 |
|  |  | 2b) Is10-Ellidavogur, Reykjanes Peninsula | 64° 07' 50.00”N 21° 50' 43.00”W | S1:9  S4:4 |
| 3 | Shetland (SH) |  | 60o 14’ 31.20”N 01o 22’ 40.68”W | S4:4 |
| 4 | Skagerrak (Sk) |  | 58° 19′ 24″ N  11° 32′ 49.2″ E | S4:8 |
| 5 | Orkney (OK) |  | 58° 56’ 31.35”N 3° 5’ 22.15”W | S1:23 |
| 6 | North Uist (NU) | Bagh a Chaise, Sound of Harris | 57° 38' 47.81"N    07° 04' 42.29"W | S1:10  S4:2 |
|  |  | Loch Blathaisbhal | 57° 37' 19.33"N 07° 11' 48.23"W | S1:1  S4:2 |
|  |  | Traigh Athmor IT1 | 57° 38' 28.20"N 07° 12' 59.28"W | S1:9 |
|  |  | Loch Maddy Harbour Core | 57° 35' 52.43'' N 07° 09' 05.01'' W | S4:3 |
| 7 | Ythan Estuary (YN) |  | 57°20’N, 01°57’W | S1:9  S5:20 |
| 8 | Baltic sea, (BA) | 8a) C-An-1-normal salinity, Anholt | 56° 26’ 02.88”N 11° 50’ 02.58”E | S4:2 |
|  |  | 8b) C-Ha-1-low salinity, Hanӧ Bay | 55° 38' 00.00”N 14° 50’ 00.00”E | S4:8 |
| 9 | Eden Estuary (ED) |  | 56°22’ 00.00”N 02°50’.00W | S1:74 |
| 10 | Cramond (CD) |  | 55° 59' 22.92''N 03° 17' 53.16''W | S1:3  S5:7 |
| 11 | Loch na Cille (LK) |  | 55° 57’ 36.00”N 05° 41’ 24.00”W | S1:14 |
| 12 | White Rock (WR) |  | 54° 29’ 05.42”N 05° 39’ 12.58”W | S1:16 |
| 13 | Cork (CK) | Timoleague, County Cork | 51° 38' 29.40''N 08° 45' 44.50''W | S1:13 |
|  |  | Ring, County Cork | 51° 36’ 39.50”N 08° 51’ 14.00”W | S1:9 |
| 14 | Aberdovey Marshes (AB) |  | 52° 31' 45.01'' N 04° 00' 07.06'' W | S1:95  (Genotyped: 18,  SEM imaged:77)  *E. williamsoni* holotype:1  *E. williamsoni* paratype :10 |
| 15 | Dartmouth England (DM) |  | 50° 21’ 04.84”N 03° 34’ 11.33”W | S1:24  S5:10 |
| 16 | Baie de Seine (BS) |  | 49° 31′ 50.40″N 00° 01′ 06.18″E | S5:2 |
| N/A | N/A |  | N/A | *Polystomella umbilicatula*: 25 |
|  |  |  | Total number of specimens analysed | 419 |
